# Authors’ reply

**Published:** 2010

**Authors:** Renu B. Pattanshetty, G. S. Gaude

**Affiliations:** **From:** KLE Institute of Physiotherapy, Belgaum, India

Dear Editor,

We thank the author[1] for his appreciation and interest shown toward our publication.[2]

Clinical diagnosis based on new pulmonary opacity and purulent respiratory secretions, and other signs of inflammation is extremely valuable in detecting patients with ventilator associated pneumonia (VAP). As mentioned in our article,[2] the baselines values are mentioned in both the groups which did show CPIS values >6. Though CPIS score may have limited specificity and sensitivity,[3] it has shown to be extremely useful for clinicians to direct the medical and antibiotic therapy.[4] The pretest probability of development of VAP has been measured by CPIS and the score has established the likelihood that patient has VAP. Serial versions have also been used to establish the clinical resolution of VAP.[5] Also, use of CPIS may be of much help to the clinicians for a patient-based approach for appropriate antibiotic therapy.[4] Hence, using CPIS score for diagnosis and treatment of VAP was selected as the most appropriate evaluating criterion.

The present study mentions that ventilated patients in the ICU frequently have impaired mucus transport which is associated with the development of retention of secretions and pneumonia, and chest physiotherapy has a role to manage the clearance of the trachea–bronchial airway, which in turn may prevent VAP. The authors completely agree to the fact that evidence base for chest physiotherapy for prevention of VAP is extremely limited.[6] The current study has shown that multimodiality chest physiotherapy may be helpful in decreasing the mortality rate and also in prevention of VAP. However, the results of the trial need to be confirmed by using a larger sample size, similar ICU set-ups, and multicentre patient enrolment.

Regarding semi-recumbent positioning (head-end elevation to 30°–45°) and prevention of VAP, there is enough evidence to suggest that semi-recumbent positioning is one of the important strategies for prevention of VAP.[7,8] Evidence also suggests that semi-recumbent position should be used routinely to reduce the risk of VAP,[9] nevertheless, to say that physiotherapy intervention in intensive care is safe.[10]

The present study was single blinded with random allocation of all patients in the trial to either of the two groups; hence, the authors suspect no bias or differences in the mortality rate reported in the study, attributable to study design. However, the authors agree that APACHE scores have not been considered since patients treated with multimodiality chest physiotherapy were referred by the physician/surgeon, considering their hemodynamic stability and disease severity. However, it may be considered as a valuable suggestion for future trials.

The inaccuracy present in the body of the text (Jessica *et al*. should have been Choi *et al*.) is regretted.

The study was aimed to find out the effect of multimodality chest physiotherapy and its role in prevention of VAP using overall CPIS score as one of the outcome measures. We did not aim to study the treatment outcome of VAP; hence, the microbiological results were not discussed in the current study. The treatment was left to the discretion of the treating physician.

However, the present randomized controlled trial may be considered as an important addition as one of the preventive strategies to reduce VAP.
